# Comparative analysis of the primary structure and production of recombinant poly(ADP-ribose)polymerase 1 of long-lived Heterocephalus glaber

**DOI:** 10.18699/vjgb-24-77

**Published:** 2024-11

**Authors:** K.N. Naumenko, A.R. Nurislamov, K.D. Nazarov, V.S. Fishman, A.A. Popov, I.O. Petruseva, A.N. Evdokimov, O.I. Lavrik

**Affiliations:** Institute of Chemical Biology and Fundamental Medicine of the Siberian Branch of the Russian Academy of Sciences, Novosibirsk, Russia; Institute of Cytology and Genetics of the Siberian Branch of the Russian Academy of Sciences, Novosibirsk, Russia; Institute of Chemical Biology and Fundamental Medicine of the Siberian Branch of the Russian Academy of Sciences, Novosibirsk, Russia; Institute of Cytology and Genetics of the Siberian Branch of the Russian Academy of Sciences, Novosibirsk, Russia; Institute of Chemical Biology and Fundamental Medicine of the Siberian Branch of the Russian Academy of Sciences, Novosibirsk, Russia; Institute of Chemical Biology and Fundamental Medicine of the Siberian Branch of the Russian Academy of Sciences, Novosibirsk, Russia; Institute of Chemical Biology and Fundamental Medicine of the Siberian Branch of the Russian Academy of Sciences, Novosibirsk, Russia; Institute of Chemical Biology and Fundamental Medicine of the Siberian Branch of the Russian Academy of Sciences, Novosibirsk, Russia

**Keywords:** poly(ADP-ribose)polymerase 1, DNA repair, Heterocephalus glaber, поли(ADP-рибоза)полимераза 1, репарация ДНК, Heterocephalus glaber

## Abstract

DNA repair is a most important cellular process that helps maintain the integrity of the genome and is currently considered by researchers as one of the factors determining the maximum lifespan. The central regulator of the DNA repair process is the enzyme poly(ADP-ribose)polymerase 1 (PARP1). PARP1 catalyzes the synthesis of poly(ADP-ribose) polymer (PAR) upon DNA damage using nicotinamide adenine dinucleotide (NAD+) as a substrate. This polymer covalently attaches to PARP1, which leads to its dissociation from the complex with damaged DNA and stimulation of the repair process. Despite intensive research on PARP1, its properties as an isolated protein have not been practically studied in mammals that demonstrate a long maximum lifespan, such as, for example, the naked mole rat (Heterocephalus glaber). High activity of DNA repair systems is observed in the cells of the naked mole rat, which ensures their high resistance to oxidative stress, as well as to genotoxic effects. The revealed features may be due to the high activity of PARP1 in the cells of the naked mole rat; however, this issue remains poorly understood and, thus, requires more detailed research, including one with the use of isolated protein PARP1 of the naked mole rat, the isolation and characterization of which have not been carried out before. In the present work, the amino acid sequence of PARP1 of the naked mole rat is compared with the amino acid sequences of orthologous proteins of other mammals. In contrast to human PARP1, 13 evolutionarily conservative amino acid substitutions in various functional domains of the protein have been identified in the amino acid sequence of naked mole rat PARP1. Using the cDNA of the naked mole rat’s Parp1 gene, a vector was created for the expression of the target protein in Escherichia coli cell culture. For the first time, a detailed description of the procedure for the expression and purification of the recombinant protein PARP1 of the long-lived naked mole rat is presented. In addition, poly(ADP-ribose)polymerase activity of the obtained protein was evaluated. The results presented in this paper are the basis for further detailed characterization of the properties of purified recombinant naked mole rat PARP1.

## Abstract (ru)

Репарация ДНК – важнейший клеточный процесс, который способствует поддержанию целостности генома. В настоящее время эффективная работа систем репарации ДНК рассматривается исследователями как один из ключевых факторов, определяющих максимальную продолжительность жизни. Центральным регулятором процесса репарации ДНК является фермент поли(ADP-рибоза)полимераза 1 (PARP1), способный синтезировать полимер поли(ADP-рибозы) (PAR) в ответ на повреждение ДНК и присоединять его к белкам- мишеням, в число которых входит и сам PARP1, осуществляя тем самым посттрансляционную модификацию этих белков и регулируя их сродство к ДНК. PARP1 принимает участие и во многих других процессах, ассоциированных с клеточным старением, таких как поддержание целостности теломер и развитие воспалительной реакции. Свойства PARP1 как изолированного белка практически не исследовались у млекопитающих, кото- рые демонстрируют высокую максимальную продолжительность жизни, за исключением человека. Одним из перспективных объектов таких исследований считается голый землекоп (Heterocephalus glaber), имеющий экстремально высокую максимальную продолжительность жизни, а также более эффективно функционирую- щие системы репарации ДНК, которые обеспечивают высокую устойчивость его клеток к воздействию ряда генотоксических агентов, по сравнению с другими мелкими грызунами, например, близкой по размеру и массе тела мышью (Mus musculus). В настоящей работе проведено сравнение аминокислотной последовательности PARP1 голого землекопа с аминокислотными последовательностями белков-ортологов других млекопитаю- щих. В отличие от PARP1 человека, в аминокислотной последовательности PARP1 голого землекопа выявлено 13 эволюционно консервативных аминокислотных замен в различных функциональных доменах белка. С ис- пользованием поиска в базах данных последовательности кДНК гена Parp1 голого землекопа и последующего анализа путем выравнивания транскриптомных данных выбрана соответствующая экспрессируемому варианту Parp1 последовательность кДНК, которая была клонирована с помощью экспрессионного вектора на основе плазмиды pLate31. В результате экспрессии в штамме Escherichia coli BL21(DE3)GeneX и очистки, проведенной с использованием трех хроматографических стадий, впервые был получен и охарактеризован функционально активный фермент PARP1 голого землекопа.

## Introduction

Genomic instability is a principal contributor to aging, arising from the accumulation of DNA damage due to exogenous and endogenous factors (López-Otín et al., 2023). DNA repair mechanisms are crucial in mitigating these detrimental effects and preserving genome integrity. The efficiency of DNA repair systems is linked to increased longevity (Schumacher et al., 2021). Consequently, current research focuses on elucidating the mechanisms and functionality of DNA repair systems in mammalian cells with notable life expectancies.

A particularly promising model for such research is the naked mole rat (Heterocephalus glaber), which exhibits a significantly longer lifespan compared to the mouse (Mus musculus) of similar size and body weight (Buffenstein, 2005; Gorbunova et al., 2014). Comparative studies have demonstrated that naked mole rat cells exhibit heightened DNA repair activity and increased resistance to certain genotoxic agents (methane methyl sulfonate, paraquat, etoposide, etc.) relative to mouse cells (Salmon et al., 2008; MacRae et al., 2015; Evdokimov et al., 2018, 2021). To uncover the underlying reasons for these attributes, studies on isolated mole rat proteins involved in DNA repair are imperative.

Poly(ADP-ribose) polymerase 1 (PARP1) is the central regulator of DNA repair in mammalian cells, catalyzing the synthesis of poly(ADP-ribose) (PAR) using NAD+ as a substrate. This process includes auto-poly(ADP-ribosyl) ation (autoPARylation) and the modification of other acceptor proteins through trans-poly(ADP-ribosyl)ation. PARP1 modulates the activity of repair enzymes, their interaction with damaged DNA, and their recruitment to DNA damage sites (Sinha et al., 2021; Bilkis et al., 2023; Rouleau-Turcotte, Pascal, 2023). Furthermore, PARP1 contributes to the formation of non-membrane compartments, concentrating damaged DNA and repair proteins to enhance the DNA repair process (Singatulina et al., 2019; Leung, 2020; Alemasova, Lavrik, 2022). These functions signify that PARP1 is a crucial factor in regulating DNA repair efficiency and ensuring genomic stability.

An early study examining this relationship found a positive correlation between PARP activity and lifespan across thirteen mammalian species, with humans exhibiting the longest maximum lifespan (Grube, Bürkle, 1992). Subsequent studies of the PARylation reaction kinetics, catalyzed by recombinant PARP1 from humans (Homo sapiens) and short-lived gray rats (Rattus norvegicus), indicated superior efficiency of the human enzyme (Beneke et al., 2000, 2010).

Additionally, comparative studies of DNA repair efficiency in naked mole rat and mouse cells revealed higher PAR synthesis activity and greater PARP1 protein levels in naked mole rat cells, as evidenced by covalent DNA attachment (Evdoki-mov et al., 2018). Investigating the properties of naked mole rat PARP1, and comparing them with those from other species, is therefore of significant interest. However, prior studies have not examined naked mole rat PARP1 as an isolated protein due to the unavailability of individual protein preparations.

This study aims to obtain recombinant naked mole rat PARP1 in order to investigate its properties and compare them with PARP1 from other mammals. To achieve this, a comparative analysis of the amino acid sequence of naked mole rat PARP1 with orthologous proteins from other mammals was conducted. Bioinformatics analysis identified the cDNA sequence corresponding to the expressed variant of the naked mole rat Parp1 gene, which was subsequently cloned into the pLate31 plasmid vector. This facilitated the expression, purification, and characterization of recombinant PARP1 from this long-lived rodent in E. coli cells for the first time.

## Materials and methods

Oligodeoxynucleotides. The sequences of the oligodeoxynucleotides used in this study are presented in the Table. Oligodeoxynucleotides 1–3 were synthesized at the Laboratory of Synthetic Biology, Institute of Chemical Biology and Fundamental Medicine of the Siberian Branch of the Russian Academy of Sciences, Novosibirsk, Russia (Novosibirsk, Russia). Primers pLate31-PARP1-For and pLate31-PARP1-Rev were synthesized by DNA-Sintez LLC (Moscow, Russia).

**Table 1.**
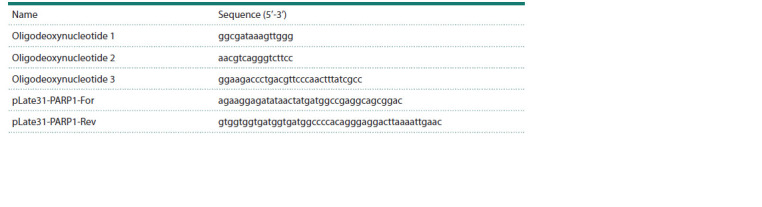
Sequences of oligodeoxynucleotides and primers used in the work

Evolutionary analysis of the primary structure of naked mole rat PARP1. To identify amino acid substitutions unique to naked mole rat PARP1, multiple sequence alignments of PARP1 amino acid sequences from this species and other mammals were conducted. The analysis included PARP1 orthologs from ten animal species: three rodents (Fukomys damarensis, M. musculus, R. norvegicus) and six other mammals (H. sapiens, Equus caballus, Dasypus novemcinctus, Loxodonta africana, Monodelphis domestica, Ornithorhynchus anatinus). PARP1 amino acid sequences were retrieved from the NCBI and Ensembl databases. Multiple alignment was performed using Clustal Omega (https://www.ebi.ac.uk/ jdispatcher/msa/clustalo).

Cell cultivation. Naked mole rat skin fibroblasts (NSF8 line) were cultured in αMEM medium supplemented with 15 % FBS, 10 % AmnioMAX, 0.005 μg/ml bFGF, and an antibiotic/antimycotic mixture (Gibco, USA) at 32 °C and 5 % CO2.

Isolation of total RNA from naked mole rat fibroblasts and preparation of cDNA. The resulting cell culture was washed with 5 ml of PBS to remove any remaining medium and 1 ml of TRIzol solution (Thermo Fisher Scientific, USA) was added. The cells were resuspended to a homogeneous suspension and transferred to a clean tube. 200 μl of chloroform was added, the tube was incubated for 5 minutes at room temperature and centrifuged for 15 minutes at 16,000 g at 4 °C. After centrifugation, the upper aqueous phase was collected into a clean test tube. The resulting sample was reprecipitated with isopropyl alcohol. The precipitated RNA was dissolved in 200 μl of water and an equal volume of phenol : chloroform : isoamyl alcohol (25 : 24 : 1) was added. After centrifugation, the aqueous phase was collected and the RNA was reprecipitated with ethanol.

To produce Parp1 cDNA, a reaction mixture with a volume of 10 μl containing 4 μg of total RNA and 100 nmol of oligo dT was incubated for 2 min at 70 °C, after which the reverse transcription buffer and 1 μl of RT-MMLV reverse transcriptase (100 units of activity/μl; Biolabmix, Russia) were added. The reaction was carried out for an hour at 42 °C.

Construction of a PARP1 expression vector. A PCR product encoding the translated region of naked mole rat PARP1 cDNA, flanked by specific nucleotide sequences, was generated using the primer pair pLate31-PARP1-For/ pLate31-PARP1-Rev (see the Table). The product was reprecipitated with 96 % ethanol, dissolved in 10 μl of LIC buffer, and treated with 1 μl of T4 phage DNA polymerase (1 U/μl; Thermo Fisher Scientific, USA). After thorough mixing and a 5-minute incubation at room temperature, the reaction was halted by adding EDTA to a final concentration of 50 mM. Subsequently, 20 fmol of the linearized pLate31 vector (Thermo Scientific, USA) with complementary “sticky” ends was added, mixed, and the mixture was incubated for 5 minutes at room temperature. The resulting plasmid DNA was used to transform E. coli XLBlue cells.

Determination of the total level of poly(ADP-ribose) synthesized in the autoPARylation reaction. Reaction mixtures (10 μl) containing 100, 200, or 400 nM recombinant PARP1 protein, 100 nM 32 bp DNA duplex with a singlestrand break (from hybridization of oligodeoxynucleotides 1–3, see Table 1), 400 μM NAD+, and [32P]-labeled NAD+ (0.4 μCi) were prepared. The reaction commenced with the addition of NAD+ and the mixtures were incubated at 37 °C for 10 minutes. The reaction was terminated by applying the mixture to chromatographic paper (GE Healthcare, USA) pre-treated with 10 % TCA. Unincorporated [32P]-NAD+ was removed through successive washes with 5 % TCA and ethanol. The paper was dried, and PAR synthesis levels were assessed via autoradiography using Typhoon FLA 7000 (GE Healthcare, USA).

Isolation and purification of recombinant PARP1 protein. E. coli BL21(DE3)GeneX cells transformed with the pLate31-PARP1 plasmid were incubated in an autoinduction system (Studier method) using LB medium containing 50 mM Na2HPO4, 50 mM KH2PO4, 25 mM (NH4)2SO4, 2 mM MgSO4, 0.5 % glycerol, 0.05 % glucose, 0.2 % lactose, and ampicillin (100 μg/ml) for 18 hours at 37 °C. Post-incubation, cells were pelleted by centrifugation at 3,000 g, the supernatant was collected, and the cell pellet was stored at –70 °C.

For cell lysate preparation, the biomass was resuspended in buffer (20 mM Tris-HCl pH 8.0, 10 % glycerol, 2 mM 2-mercaptoethanol, 10 mM imidazole, 0.5 mM PMSF, protease inhibitors) at 5 ml buffer per 1 g cell biomass. After 20 minutes on ice, an equal volume of buffer (20 mM Tris- HCl pH 8.0, 2 M NaCl, 2 % NP-40, 10 % glycerol, 2 mM 2-mercaptoethanol, 0.5 mM PMSF, protease inhibitors) was added. The suspension was ultrasonically processed at 40 kHz for 20 minutes at 4 °C, followed by centrifugation at 30,000 g for 30 minutes in a Beckman JA 25.50 rotor.

The clarified lysate was passed through a Ni-NTA-agarose column (GE Healthcare, USA), equilibrated with buffer (20 mM Tris-HCl pH 8.0, 1 M NaCl, 2 mM 2-mercaptoethanol, 10 % glycerol, 5 mM imidazole). The column was washed with equilibration buffer and subsequently with buffer (20 mM Tris-HCl pH 8.0, 0.1 M NaCl, 2 mM 2-mercaptoethanol, 10 % glycerol, 5 mM imidazole) until baseline stabilization. Elution was performed using 250 mM imidazole buffer. Fractions containing the target protein were pooled and applied to a heparin-Sepharose column (GE Healthcare, USA). Chromatographic separation was performed isocratically with 0.3 M NaCl buffer for washing off weakly bound proteins and 1 M NaCl buffer for eluting the target protein. Fractions with the target protein were pooled and diluted 10-fold with buffer (20 mM Tris-HCl pH 8.0, 7 mM 2-mercaptoethanol, 10 % glycerol) before application to an ssDNA cellulose column (Sigma, USA). Chromatographic separation on ssDNA cellulose followed similar conditions to heparin- Sepharose separation.

## Results and discussion

**Fig. 1.**
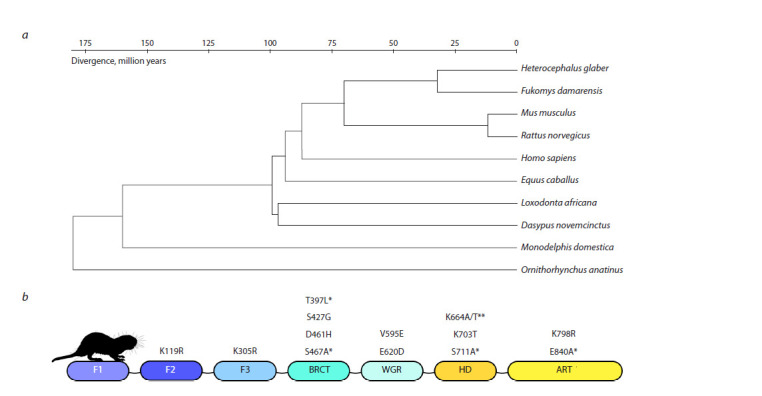
Evolutionary analysis of the primary structure of the PARP1 protein. а – list of mammal species included in the analysis and their divergence; b – amino acid substitutions in PARP1 unique to the naked mole rat. * – substitutions also present in the Damaraland mole rat; ** – substitution options from the NCBI and Ensembl databases.

To determine whether naked mole rat PARP1 contains evolutionarily conserved amino acid substitutions that could influence its functional properties, we compared its amino acid sequence with those of orthologous proteins in other mammals. The PARP1 sequence is highly conserved among mammals, demonstrating over 90 % homology despite divergence over 150 million years (Fig. 1a). Naked mole rat PARP1 retains all functional domains found in other mammals, though several substitutions were identified in highly conserved functional domain sites (Fig. 1b). Some of these substitutions also appear in F. damarensis, a related species in the Bathyergidae family. These substitutions may impact PARP1’s ability to recognize damaged DNA and its catalytic functions.

As a result of searching the cDNA sequence of the naked mole rat Parp1 gene in databases and subsequent analysis by aligning transcriptomic data for various organs of the naked mole rat (brain: SRS899007; testicles: SRR1959204; liver: ERS1090459) on three alternative Parp1 templates, the cDNA sequence (NCBI NM_001310226.1 (Bens et al., 2016)) corresponding to the expressed variant of Parp1 was chosen. We selected this cDNA for amplification and subsequent cloning.

To produce recombinant naked mole rat PARP1 in E. coli cells, an expression vector based on the pLate31 plasmid (Thermo Scientific, USA) was used. Specific primers and total cDNA from naked mole rat fibroblasts were used to amplify the coding sequence of PARP1 via PCR. The PCR product was annealed with the linearized pLate31 vector, and E. coli XLBlue cells were transformed to amplify plasmid DNA. The absence of errors in the amplified sequence was confirmed by Sanger sequencing.

**Fig. 2.**
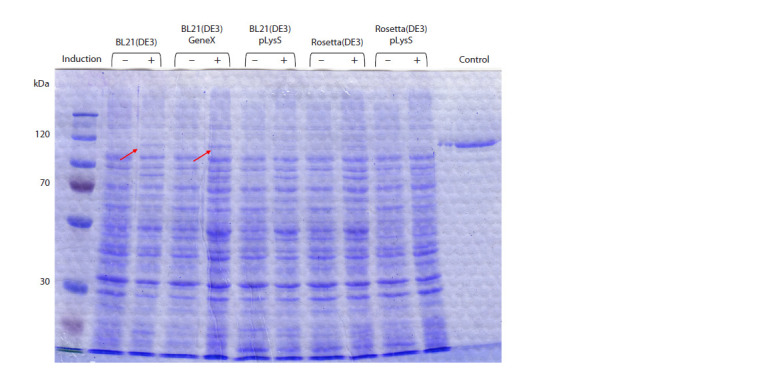
Analysis of PARP1 content in lysates of transformed E. coli cells. Cells were cultured in an autoinduction system at 37 °C with and without lactose induction. The position of the PARP1 protein is indicated by red arrows. Control – recombinant human PARP1 protein.

When searching for optimal conditions, various E. coli strains (BL21(DE3), BL21(DE3)plysS, BL21(DE3)GeneX, Rosetta(DE3), and Rosetta(DE3)plysS) were tested for optimal PARP1 expression conditions. Target protein expression was visually detected in induced BL21(DE3) and BL21(DE3) GeneX cells (Fig. 2).

**Fig. 3.**
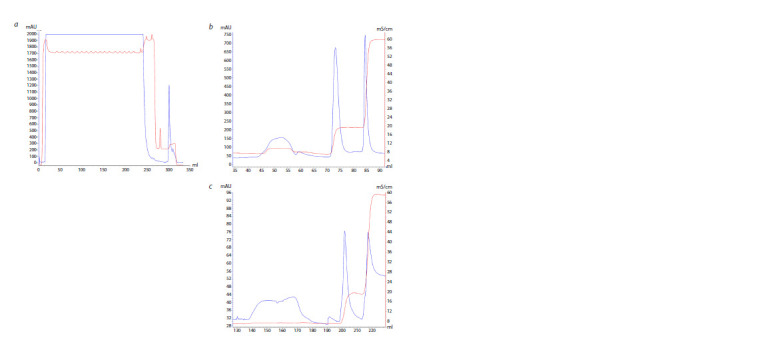
Elution profiles of the PARP1 (H. glaber) protein from Ni-NTAagarose (a), heparin-Sepharose (b), ssDNA-cellulose (c). Blue line – optical density of the solution at a wavelength of 280 nm (mAU); red line – solution conductivity (mS/cm).

The resulting cultivation conditions were used to produce a preparative amount of biomass from BL21(DE3)GeneX cells transformed with the pLate31-PARP1 vector. The resulting biomass was lysed, followed by treatment in an ultrasonic disintegrator and centrifuged to sediment debris. Next, three chromatographic purification stages were carried out sequentially using columns containing Ni-NTA (Fig. 3a), heparin-Sepharose (Fig. 3b), and ssDNA cellulose (Fig. 3c) as a sorbent (Sukhanova et al., 2004).

**Fig. 4.**
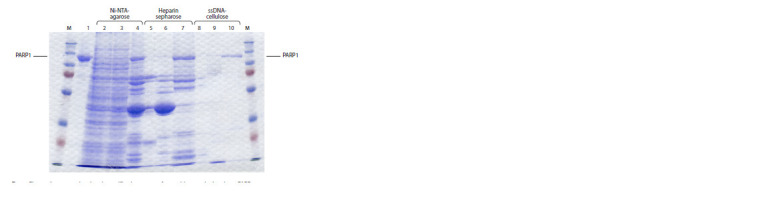
Electropherogram showing the purification stages of recombinant naked mole rat PARP1. 1 – control protein sample; 2–4 – application, breakthrough and elution from Ni-NTA-agarose, respectively; 5 – breakthrough from heparin-Sepharose; 6, 7 – elution from heparin-Sepharose 0.3 M and 1 M NaCl, respectively; 8 – breakthrough from ssDNA cellulose; 9, 10 – elution of the target protein from ssDNA cellulose with 0.3 M and 1 M NaCl, respectively.

The presence of the target protein was monitored by electrophoretic analysis with Laemmli staining (Fig. 4). Fractions containing the purified protein preparation were concentrated using Amicon 10 kDa and the purity of the purified preparation was shown by electrophoresis followed by Coomassie R250 staining.

The protein concentration in the final preparation, determined by the Bradford method, was 0.5 mg/ml. The total yield was 0.3 mg of protein per 10 g of E. coli cell biomass.

**Fig. 5.**
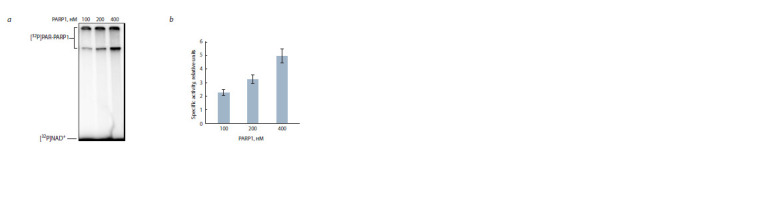
Analysis of the activity of recombinant naked mole rat PARP1 in the autoPARylation reaction. а – autoradiography of 10 % SDS-PAGE, in which the separation of protein modification products was carried out; b – diagram summarizing the results of three independent experiments performed using the TCA target method (see Materials and methods).

To test the activity of the resulting recombinant protein, we used an in vitro system containing radiolabeled NAD+ and model damaged DNA containing a break and free blunt ends as a cofactor to activate the PAR synthesis reaction catalyzed by PARP1 (Fig. 5). As can be seen from the data presented, the isolated protein has enzymatic activity in the autoPARylation reaction and is suitable for further study of its properties.

Comparative studies of DNA repair systems in naked mole rat and mouse (M. musculus) cells showed that naked mole rat cells have more effective base excision repair (BER) and nucleotide excision repair (NER) systems than mouse cells (Evdokimov et al., 2018). PARP1 activity was also significantly higher in long-lived naked mole rat cells compared to short-lived mouse cells (Evdokimov et al., 2018). Future work will involve determining the nature of the interaction between isolated naked mole rat PARP1 and partner proteins in DNA repair, the influence of these proteins on PARP1 activity, and PARP1’s affinity for damaged DNA.

Production of recombinant proteins like PARP1 requires selecting optimal conditions for production, isolation, and purification, which can differ from standard methods. The described procedures enabled successful cloning, production in the E. coli expression system, and chromatographic purification of naked mole rat PARP1. The proposed chromatographic protein purification procedure, including Ni-NTA chromatography and two “pseudo-affinity” columns under specific buffer and salt conditions, can effectively purify recombinant naked mole rat PARP1.

The comparative analysis of naked mole rat PARP1 and human ortholog protein revealed that substitutions in the ART domain did not affect the catalytic triad, suggesting similar kinetic parameters for PAR synthesis by these enzymes. However, substitutions in other functional domains, particularly those involving changes in residue type near autoPARylation targets (e. g., K305R in the Zn3 domain, D461H in the BRCT domain), may affect the protein’s properties. Further studies using mutant forms of naked mole rat PARP1 with these amino acid substitutions are warranted.

## Conclusion

Studying the properties of PARP1 in various long-lived mammals is a promising area of research, as it may contribute to a deeper understanding of the role of DNA repair in aging and how this process is organized in mammalian cells.

Comparison of PARP1 amino acid sequences of two mammals with a long maximum lifespan, the naked mole rat and the human, revealed 13 evolutionarily conserved substitutions in the naked mole rat protein. The impact of these substitutions on the properties and functions of PARP1 remains to be determined. Additionally, cloning of naked mole rat PARP1 was carried out for the first time, along with its production in E. coli cells and purification of the recombinant protein using a relatively simple procedure. The recombinant protein’s ability to perform the autoPARylation reaction was also assessed. Future work will compare the properties of recombinant PARP1 in long-lived naked mole rats and humans, a task, which has not been previously undertaken.

## Conflict of interest

The authors declare no conflict of interest.
